# Peritoneal metastatic gastric carcinoma cells exhibit more malignant behavior when co-cultured with HMrSV5 cells

**DOI:** 10.18632/aging.102803

**Published:** 2020-02-22

**Authors:** Lijing Wang, Zhiyuan Xu, Can Hu, Shangqi Chen, Yian Du, Ling Huang, Chengwei Shi, Shaowei Mo, Xiangdong Cheng

**Affiliations:** 1 Department of Ultrasound, Institute of Cancer and Basic Medicine, Cancer Hospital of the University of Chinese Academy of Sciences; Zhejiang Cancer Hospital, Hangzhou 310022, Zhejiang, China; 2 Department of gastric surgery, Institute of Cancer and Basic Medicine, Cancer Hospital of the University of Chinese Academy of Sciences; Zhejiang Cancer Hospital, Hangzhou 310022, Zhejiang, China; 3 Department of Gastrointestinal Surgery, The First Affiliated Hospital of Zhejiang Chinese Medical University, Hangzhou 310000, Zhejiang, China

**Keywords:** peritoneal metastasis, gastric cancer, NUGC-4 cells

## Abstract

Metastasis and recurrence are major causes of death in gastric cancer patients. Because there are no obvious clinical symptoms during the early stages of metastasis, we sought to isolate highly invasive metastatic gastric cancer cells for future drug screening. We first established a mouse model to observe gastric cancer metastasis *in vivo*. The incidence of peritoneal metastasis of gastric cancer was much higher than liver or lymph metastasis. Peritoneal metastatic and non-metastatic NUGC-4 cells were isolated from the mouse model. Cell proliferation was measured using CCK-8 assays, while migration and invasion were investigated in Transwell assays. Proteins involved in epithelial-mesenchymal transition were detected by Western blotting. Metastatic gastric carcinoma cells were more proliferative and invasive than primary NUGC-4 cells. The supernatants of metastatic gastric carcinoma cells notably altered the morphology of HMrSV5 peritoneal mesothelial cells and promoted their epithelial-mesenchymal transition. Moreover, primary or metastatic gastric cancer cells co-cultured with HMrSV5 cells markedly increased cancer cell proliferation and invasiveness. Moreover, peritoneal metastatic gastric carcinoma cells in the presence of HMrSV5 cells exhibited most malignant behaviors. Thus, peritoneal metastatic gastric carcinoma cells exhibited high capacities for proliferation and invasion, and could be used as a new drug screening tool for the treatment of advanced gastric cancer and peritoneal metastatic gastric cancer.

## INTRODUCTION

Gastric cancer is the third-leading cause of cancer death worldwide [1]. It is estimated that 961,000 new cases of gastric cancer are diagnosed annually [2]. Although the exact cause of gastric cancer is unclear, its pathogenesis is the same as those of other malignant tumors [3]. Gastric cancer cases can be divided into early- and advanced-stage gastric cancer [4], and the stage of the tumor determines the treatment strategy and effectiveness. Early- stage gastric cancer patients typically undergo radical surgery followed by chemotherapy, and the postoperative five-year survival rate is 90% [5]. Advanced-stage gastric cancers include intermediate and advanced tumors [4]. Despite rapid progress in gastric cancer research, there is still no effective treatment for advanced gastric cancer.

Peritoneal metastasis is the most common form of metastasis in gastric cancer [6], though it is not yet known why peritoneal diffusion is favored. The incidence of peritoneal metastasis increases with the depth of tumor invasion. The metastasis of gastric cancer cells to the peritoneum usually proceeds along peri-gastric regions to the branches of the celiac trunk [7]. Surgery and chemotherapy can somewhat alleviate the pain caused by the peritoneal metastasis of gastric cancer [8]. However, the five-year relapse-free survival rate for peritoneal metastatic gastric cancer patients is only 53% [6].

The peritoneal metastasis of gastric cancer involves the interaction between gastric cancer cells and peritoneal mesothelial cells. Gastric cancer cells can promote the fibrosis and migration of mesothelial cells, which in turn prepare the peritoneum for the adhesion and invasion of gastric cancer cells [9–13]. Based on this background, we sought to isolate highly invasive metastatic gastric cancer cells for future use as drug screening tools for the treatment of advanced gastric cancer.

## RESULTS

### The peritoneum and liver were the most common sites of gastric cancer cell metastasis in mice

**Figure 1:**
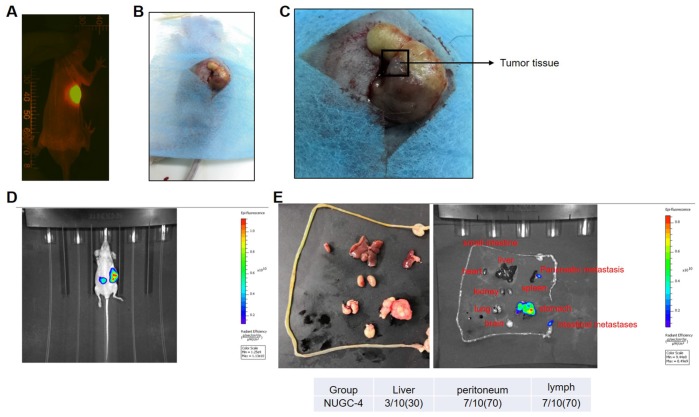
**A mouse model of gastric carcinoma was successfully established.** (**A**) A nude mouse model of human gastric carcinoma was constructed through orthotopic transplantation. The gastric orthotopic model was observed with an In Vivo Image System. (**B**, **C**) The gastric orthotopically transplanted mice were sacrificed at the end of study, and their gastric tissues are pictured. (**D**) Tumor metastasis in the mice was detected with an In Vivo Image System eight weeks after gastric orthotopic transplantation. (**E**) The heart, liver, spleen, lung, kidney, small intestine, peritoneum, lymph and brain tissues of the mice were isolated and photographed eight weeks after gastric orthotopic transplantation.

In order to isolate highly invasive metastatic gastric cancer cells *in vivo*, we first established a mouse model of gastric carcinoma (Figure 1A–1D). As shown in Figure 1E, peritoneal, liver and lymph metastases of gastric cancer were notably more prevalent than metastases to other parts of the mice.

### Metastatic gastric cancer cells exhibited more malignant behavior than primary gastric cancer cells *in vitro*

**Figure 2:**
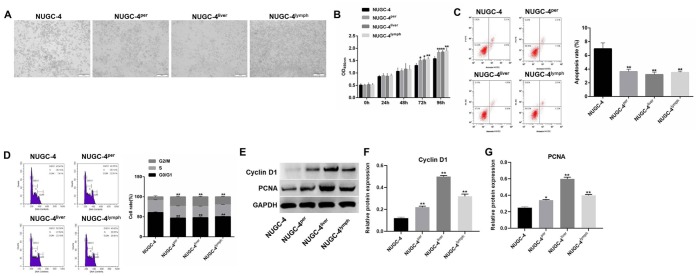
**Metastatic gastric cancer cells exhibited more malignant behavior than primary gastric cancer cells *in vitro*.** (**A**) The morphology of primary or metastatic NUGC-4 cells was observed under a microscope. (**B**) A CCK-8 assay was used to detect the viability of NUGC-4 (control), peritoneal metastatic NUGC-4 (NUGC-4^per^), liver metastatic NUGC-4 (NUGC-4^liver^) and lymph metastatic NUGC-4 (NUGC-4^lym^) cells at 0, 24, 48, 72 and 96 h. (**C**) A flow cytometer was used to detect the apoptosis of NUGC-4, NUGC-4^per^, NUGC-4^liver^ and NUGC-4^lym^ cells that had been incubated for 48 h. (**D**) A flow cytometer was used to detect the cell cycle distribution of NUGC-4, NUGC-4^per^, NUGC-4^liver^ and NUGC-4^lym^ cells that had been incubated for 48 h. (**E**–**G**) The relative levels of Cyclin D1 and PCNA in primary or metastatic gastric cancer cells were detected by western blotting. ^*^P<0.05, ^**^P<0.01 compared to the control group.

Since peritoneal, liver and lymph metastases of gastric cancer exhibited high incidence rates *in vivo*, these three types of metastatic cells were isolated for subsequent experiments. The proliferative abilities of metastatic and primary gastric cancer cells were investigated by microscopy and a CCK-8 assay. As indicated in Figure 2A and 2B, metastatic gastric cancer cells exhibited significantly greater viability than primary gastric cancer cells.

Next, to explore the tumorigenic potential of metastatic and primary gastric cancer cells, we used a flow cytometer to evaluate the apoptosis rate and cell cycle distribution of each cell line. The apoptosis rate was much lower in metastatic gastric cancer cells than in primary cancer cells (Figure 2C). In addition, cell cycle analysis illustrated that the proportions of cells in S phase and G2/M phase were much higher among metastatic gastric cancer cells than among primary gastric cancer cells (Figure 2D). Thus, metastatic gastric cancer cells exhibited more malignant behavior than primary gastric cancer cells *in vitro*.

Finally, to explore the mechanism by which metastatic gastric cancer cells exhibited significant proliferative ability, western blot was performed. As showed in Figure 2E–2G, the protein expression of Cyclin D1 and PCNA in metastatic gastric cancer cells were much higher than those in primary gastric cancer cells. Taken together, these data suggested that metastatic gastric cancer cells exhibited significant proliferative ability via increasing the expression of Cyclin D1 and PCNA.

### Metastatic gastric cancer cells were more invasive than primary gastric cancer cells *in vitro*

**Figure 3:**
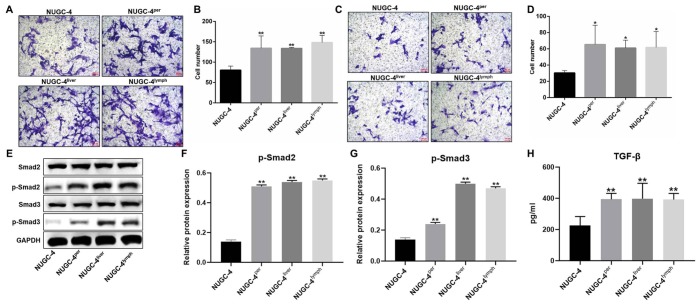
**Metastatic gastric cancer cells were more invasive than primary gastric cancer cells *in vitro*.** (**A**, **B**) NUGC-4, NUGC-4^per^, NUGC-4^liver^ and NUGC-4^lym^ cells were incubated for 48 h, and their migration was investigated by a Transwell assay. (**C**, **D**) A Transwell assay was used to investigate the invasion of NUGC-4, NUGC-4^per^, NUGC-4^liver^ and NUGC-4^lym^ cells. (**E**–**G**) The relative levels of p-Smad2 and p-Smad3 in metastatic or primary gastric cancer cells were detected by western blotting. (**H**) The levels of TGF-β in supernatants of metastatic or primary gastric cancer cells were detected by ELISA. ^*^P<0.05, ^**^P<0.01 compared to the control group.

Next, Transwell assays were used to compare the migration and invasion abilities of metastatic and primary gastric cancer cells. The migrating cell number was markedly greater for metastatic gastric cancer cells than for primary cancer cells (Figure 3A and 3B). Moreover, metastatic gastric cancer cells were significantly more invasive than primary gastric cancer cells (Figure 3C and 3D).

To explore the mechanism by which metastatic gastric cancer cells exhibited more invasive behaviors than primary gastric cancer cells, western blot was used. The results showed that the expression of p-Smad2 and p-Smad3 in metastatic gastric cancer cells was obviously higher than those in primary gastric cancer cells (Figure 3E–3G). Consistently, the levels of TGF-β in supernatants of metastatic gastric cancer cells were greatly higher than those in supernatants of primary gastric cancer cells (Figure 3H). All these results showed that metastatic gastric cancer cells exhibited more invasive behaviors via activation of TGF-β signaling.

### Metastatic gastric cancer cell supernatants significantly altered the morphology of peritoneal mesothelial cells

**Figure 4:**
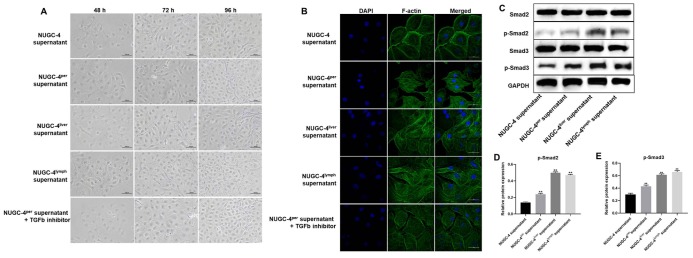
**Metastatic gastric cancer cell supernatants significantly altered the morphology of peritoneal mesothelial cells.** HMrSV5 cells were co-cultured with NUGC-4, NUGC-4^per^, NUGC-4^liver^, NUGC-4^lym^ cell supernatants and NUGC-4^per^ cell supernatants + TGF-β inhibitor for 48, 72 and 96 h. (**A**) The morphology of HMrSV5 cells was observed under an optical microscope at 48, 72 and 96 h. (**B**) DAPI and F-actin were detected by an immunofluorescence assay in HMrSV5 cells co-cultured with NUGC-4, NUGC-4^per^, NUGC-4^liver^, NUGC-4^lym^ cell supernatants and NUGC-4^per^ cell supernatants + TGF-β inhibitor for 72 h. (**C**–**E**) The relative levels of p-Smad2 and p-Smad3 in peritoneal mesothelial cells were detected by western blotting. ^**^P<0.01 compared to the control group.

We then performed an immunofluorescence assay and optical microscopy to explore the effects of metastatic gastric cancer cell supernatants on the morphology of HMrSV5 peritoneal mesothelial cells. After 72 h of co-incubation with metastatic gastric cancer cell supernatants, HMrSV5 cells became significantly more rhomboidal in morphology. However, the changes of HMrSV5 cells in the presence of peritoneal metastatic gastric cancer cell supernatants were partially reversed by TGF-β inhibitor (Figure 4A). DAPI and F-actin staining confirmed that the HMrSV5 cells exhibited a more ductile morphology (an indicator of more invasive behavior) in the presence of metastatic gastric cancer cell supernatants, while the invasive behavior of HMrSV5 cells in the presence of peritoneal metastatic gastric cancer cell supernatants were greatly rescued by TGF-β inhibitor (Figure 4B). These data suggested that metastatic gastric carcinoma cells significantly increased the invasiveness of HMrSV5 cells.

Then, to confirm the mechanism by which metastatic gastric cancer cell supernatants altered the morphology of peritoneal mesothelial cells, western blot was used. As demonstrated in Figure 4C–4E, the expressions of p-Smad2 and p-Smad3 in peritoneal mesothelial cells were significantly upregulated in the presence of metastatic gastric cancer cell supernatants. These results confirmed that metastatic gastric cancer cell supernatants altered the morphology of peritoneal mesothelial cells through activation of TGF-β pathway.

### Metastatic gastric cancer cell supernatants significantly promoted the epithelial-mesenchymal transition in peritoneal mesothelial cells

**Figure 5:**
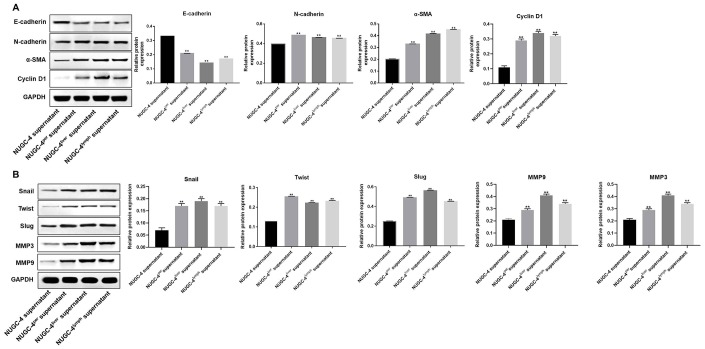
**Metastatic gastric cancer cell supernatants significantly induced the EMT in peritoneal mesothelial cells.** (**A**, **B**) HMrSV5 cells were co-cultured with NUGC-4, NUGC-4^per^, NUGC-4^liver^ and NUGC-4^lym^ cell supernatants for 72 h. Then, the relative levels of E-cadherin, N-cadherin, α-SMA, Snail, Twist, Slug, MMP3, MMP9 and Cyclin D1 in HMrSV5 cells were detected by Western blotting. ^**^P<0.01 compared to the control group.

To verify the invasiveness of the peritoneal mesothelial cells, we detected epithelial-mesenchymal transition (EMT)-related proteins by Western blotting. As shown in Figure 5A and 5B, metastatic gastric cancer cell supernatant treatment significantly downregulated E-cadherin expression in peritoneal mesothelial cells, while it notably upregulated N-cadherin, α-SMA, Snail, Twist, Slug, Cyclin D1, MMP3 and MMP9 expression. These results suggested that metastatic gastric cancer cells may have exhibited more malignant behavior than primary gastric cancer cells because they induced the EMT in peritoneal mesothelial cells.

### Peritoneal mesothelial cells significantly enhanced the proliferation of gastric cancer cells

**Figure 6:**
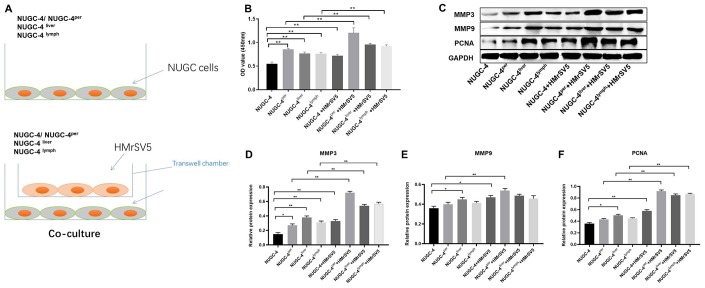
**Peritoneal mesothelial cells significantly enhanced the proliferation of gastric cancer cells.** (**A**) Primary or metastatic gastric cancer cells were co-cultured with HMrSV5 cells or nothing. (**B**) The viability of gastric cancer cells after 72 h of incubation was evaluated with a CCK-8 assay. (**C**–**F**) The relative protein expressions of MMP3, MMP9 and PCNA in primary or metastatic gastric cancer cells were detected by western blot. ^**^P<0.01.

We next performed a CCK-8 assay to determine the effects of HMrSV5 peritoneal mesothelial cells on the proliferation of primary or metastatic gastric cancer cells. As indicated in Figure 6A and 6B, peritoneal mesothelial cells significantly increased the proliferation of primary or metastatic gastric cancer cells. Moreover, peritoneal metastatic gastric carcinoma cells co-cultured with HMrSV5 cells exhibited cell proliferation to the greatest extent (Figure 6A and 6B). These data revealed that peritoneal mesothelial cells induced the proliferation of gastric cancer cells. Next, the results of western blot indicated that peritoneal mesothelial cells significantly increased the protein expressions of MMP3, MMP9 and PCNA in metastatic gastric cancer cells, especially peritoneal metastatic gastric cancer cells (Figure 6C–6F). All these data suggested that peritoneal mesothelial cells enhanced the malignant behaviors of gastric cancer cells, especially peritoneal metastatic gastric carcinoma cells.

### Peritoneal mesothelial cells significantly enhanced the invasiveness of gastric cancer cells

**Figure 7:**
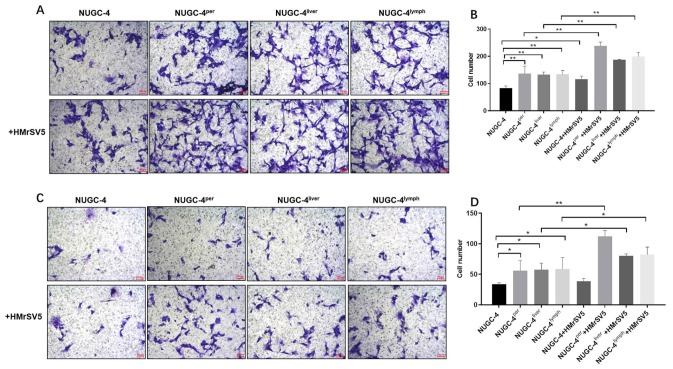
**Peritoneal mesothelial cells significantly enhanced the invasiveness of gastric cancer cells.** Primary or metastatic gastric cancer cells were co-cultured with HMrSV5 cells or nothing for 72 h. (**A**, **B**) The number of migrating primary or metastatic gastric cancer cells was determined with a Transwell assay. (**C**, **D**) The number of invading primary or metastatic gastric cancer cells was determined with a Transwell assay. ^*^P<0.05, ^**^P<0.01.

Next, we used Transwell assays to investigate the effects of peritoneal mesothelial cells on the migration and invasion of gastric cancer cells. The results indicated that peritoneal mesothelial cells notably increased the number of migrating and invasion gastric cancer cells (Figure 7A and 7B). Peritoneal metastatic gastric carcinoma cells co-cultured with HMrSV5 cells exhibited cell migration and invasion to the greatest extent (Figure 7A and 7B). The Transwell invasion assay results were consistent with the migration assay data (Figure 7C and 7D). All these data demonstrated that peritoneal mesothelial cells significantly enhanced the invasiveness of gastric cancer cells, especially peritoneal metastatic gastric carcinoma cells.

## DISCUSSION

Although great efforts have been made to improve the treatment of gastric cancer, the mortality of patients with advanced gastric cancer remains high [16–19]. In the present study, we aimed to isolate highly invasive metastatic gastric carcinoma cells for future drug screening. We found that peritoneal metastatic gastric cancer cells exhibited highly malignant behavior, indicating that they could be used as a new tool for drug screening.

We first discovered that the peritoneum and liver were the most common sites of gastric cancer cell metastasis in mice. Then, we demonstrated that peritoneal metastatic gastric cancer cells were more malignant and invasive than primary gastric cancer cells *in vitro*. Similarly, a previous study indicated that peritoneal metastatic gastric cancer cells were highly invasive [20]. Meanwhile, our findings also found that the protein expression of Cyclin D1 and PCNA in metastatic gastric cancer cells were much higher than those in primary gastric cancer cells. Cyclin D1 and PCNA were two markers of cell proliferation [21]. Wang Y et al. indicated that Genistein promoted the proliferative ability of ovarian cancer OVCAR-5 cells via upregulating the expression of Cyclin D1 and PCNA [22]. Our findings were consistent with these data, suggesting that metastatic gastric cancer cells exhibited more malignant behaviors via upregulating the expression of Cyclin D1 and PCNA.

Besides, our study also found that metastatic gastric cancer cell supernatants notably upregulated the protein expression of p-samd2 and p-smad3 in HMrSV5 cells. Smad2 and Smad3 were downstream proteins of TGF- β signaling [23]. Derynck R et al. found that TGF-β signaling played a critical role in cancer progression [24]. Our data were consistent with these results, indicating that metastatic gastric cancer cell supernatants transformed the behavior of HMrSV5 cells via activation of TGF-β signaling.

In cancer progression, the EMT is associated with increased tumor invasiveness, along with intravasations and extravasations of metastatic cancer cells [25–27]. Our experiments revealed that metastatic gastric cancer cell supernatants promoted the EMT in peritoneal mesothelial cells. Jiang et al. found that connective tissue growth factor induced the EMT in peritoneal mesothelial cells and enhanced their adhesion to gastric cancer cells [28]. We also found that HMrSV5 cells significantly increased the proliferation and migration of gastric cancer cells. Similarly, a previous study indicated that tumor necrosis factor-α-induced matrix metallopeptidase-9 secretion from mesothelial cells promoted the metastatic dissemination of gastric cancer cells [29]. Since Lyu et al. demonstrated that transforming growth factor-β1 was a key contributor to the peritoneal metastasis of gastric cancer [30], the involvement of transforming growth factor-β1 signaling in the EMT of peritoneal mesothelial cells will be further investigated. To summarize, metastatic gastric cancer cell adhesion to the peritoneum promoted the EMT in peritoneal mesothelial cells, which in turn enhanced the invasiveness of gastric cancer cells.

In conclusion, peritoneal metastatic gastric carcinoma cells exhibited greater proliferative and invasive abilities than primary cancer cells, and thus could be used as a new drug screening tool for the treatment of advanced gastric cancer.

## MATERIALS AND METHODS

### Cell lines and cell culture

The human gastric cancer cell line NUGC-4 and peritoneal mesothelial cell line HMrSV5 were purchased from the Cell Bank of the Chinese Academy of Sciences (Shanghai, China). According to Xiaojun Dai et al., NUGC-4 cells are prone to organ metastasis [29]. NUGC-4 and HMrSV5 cells were cultured in RPMI 1640 medium (Gibco, Grand Island, NY, USA) supplemented with 1% fetal bovine serum (FBS, Gibco), 100 U/mL penicillin and 100 μg/mL streptomycin (Thermo Fisher Scientific, Waltham, MA, USA). All cultures were maintained at 37°C with 5% CO_2_.

### *In vivo* experiment

Six-week-old male BALB/C nude mice (Vital River, Beijing, China) were used in this study. All mice were maintained under pathogen-free conditions. The mice were subcutaneously injected with NUGC-4 gastric carcinoma cells (5×10^6^/100 μL). After subcutaneous neoplasia developed, the tumor tissues were removed, and viable tissues were cut with scissors and minced into 2x2x2 mm^3^ pieces. These pieces were orthotopically transplanted into the stomach as previously described [29]. Orthotopic transplantation to the stomach was observed with a Retiga EXi cooled digital CCD color camera (QImaging, Surrey, BC, Canada). The images were analyzed with Image-Pro Premier (MediaCybernetics, Baltimore, MD, USA). Every three to four days, the mice were anesthetized by intraperitoneal injection of 3% sodium pentobarbital and were observed with an In Vivo Image System (IVIS, Perkin Elmer, Waltham, MA, USA). At the end of the study, the heart, stomach, liver, spleen, lung, kidney, peritoneum, small intestine and brain tissues of the mice were collected for the observation of tumor metastasis. All animal experiments were performed in accordance with the Ethics Committee of Zhejiang Cancer Hospital. We strictly followed the National Institutes of Health Guide for the Care and Use of Laboratory Animals. The IACUC approval number is “ZJCH201803048”.

### Cell isolation from metastatic foci of gastric cancer

Peritoneal metastatic NUGC-4 cells (NUGC-4^per^), liver metastatic NUGC-4 cells (NUGC-4^liver^), lymphocyte metastatic NUGC-4 cells (NUGC-4^lym^) and non-metastatic NUGC-4 cells (control) were isolated *in vitro* by a double dilution procedure [30]. All cell lines were maintained as monolayer cultures on plastic in RPMI 1640 medium (Gibco) supplemented with 1% FBS (Gibco), 100 U/mL penicillin and 100 μg/mL streptomycin (Thermo Fisher Scientific).

### Microscopy

HMrSV5 cells were co-cultured with the supernatants of NUGC-4, NUGC-4^per^, NUGC-4^liver^ and NUGC-4^lym^ cells. The morphology of the HMrSV5 cells was observed under an optical microscope (Olympus, Tokyo, Japan) after 48, 72 and 96 h.

### Cell viability evaluation

Cell viability was analyzed with the Cell Counting Kit-8 (CCK-8) assay (Beyotime, Shanghai, China). Metastatic or non-metastatic NUGC-4 cells were seeded in 96-well plates at a density of 2×10^3^ cells/well, and were incubated for 0, 24, 48, 72 and 96 h. Then, the cells were treated with 100 μL of a formazan dissolving solution for 15 min. The absorbance of the cells was determined at 450 nm on a microplate reader (Thermo Fisher Scientific).

### Cell apoptosis

Cell apoptosis was determined by flow cytometry with the Fluorescein Isothiocyanate (FITC) Annexin V Apoptosis Detection Kit I (Nanjing KeyGen Biotech Co., Nanjing, China). Briefly, cells were harvested (1×10^6^/mL), washed twice with phosphate-buffered saline (PBS) and resuspended in Binding Buffer. Then, cells (100 μL) were incubated with 5 μL of FITC Annexin V and 5 μL of propidium iodide at room temperature for 15 min in the dark. The stained cells were analyzed by flow cytometry with a Gallios instrument (Beckman Coulter, Miami, FL, USA). The percentage of apoptotic cells was quantified.

### Transwell assay

Briefly, 24-well Transwell plates (Corning, New York, NY, USA) were used for cell invasion and migration detection. For the cell migration assay, 2×10^5^ metastatic or primary NUGC-4 cells were seeded into the upper chambers of the 24-well plates in 200 μL of serum-free RPMI 1640 medium supplemented with 0.2% bovine serum albumin. The lower chambers contained RPMI 1640 medium supplemented with 1% FBS. After 24 h of incubation at 37°C, the non-migrating cells were gently removed from the upper side of each chamber with a cotton swab, while the cells that had migrated were fixed with 95% alcohol for 10 min and stained with 1% crystal violet (Sigma, Grand Island, NY, USA) for 5 min. Finally, images were captured, and the cells were counted under an inverted light microscope (Olympus) at 400x magnification.

For the invasion assay, the upper chambers of the 24-well plates were pretreated with 50 μL of Matrigel (12.5 mg/L), and the wells were pretreated with Matrigel (BD Biosciences, Franklin Lake, NJ, USA). Then, metastatic or non-metastatic NUGC-4 cells (1×10^6^ cells/mL) in FBS-free medium were seeded into the upper chambers. The lower chambers contained RPMI 1640 medium supplemented with 1% FBS. The cells were incubated at 37°C for 24 h, and cells that had attached to the underside of the membrane were fixed and stained with a 0.1% crystal violet solution. Finally, images were captured, and the number of invading cells was counted under a microscope at 400x magnification.

### Cell cycle detection

The cell cycle distribution was determined by flow cytometry with a Cycle Detection Kit I (Nanjing KeyGen Biotech Co.). Metastatic or non-metastatic NUGC-4 cells were plated in six-well plates for 72 h. Then, the cells were fixed in cold 70% ethanol at 4°C overnight and treated with 100 μL of Propidium Iodide/RNase Staining Buffer (Thermo Fisher Scientific) at room temperature in the dark for 30 min. Finally, a flow cytometer (BD Biosciences) was used to detect the cell cycle distribution.

### Western blot

Whole-cell lysates were collected in radioimmunoprecipitation assay buffer (Beyotime). Proteins were separated on 10% sodium dodecyl sulfate polyacrylamide gels and transferred to polyvinylidene difluoride membranes (Beyotime). The membranes were incubated with 5% skim milk in Tris-buffered saline-Tween at room temperature for 1 h. The membranes were then probed overnight at 4°C with the following primary antibodies (all from Affinity; 1:1000): anti-E-cadherin, anti-N-cadherin, anti-α-smooth muscle actin (SMA), anti-Snail, anti-Twist, anti-Slug anti-Cyclin D1, anti-MMP3, anti-MMP9, anti-PCNA, anti-Smad2, anti-Smad3 and anti-GAPDH. The membranes were subsequently incubated with an anti-rabbit secondary antibody (Affinity; 1:5000) at room temperature for 1 h. Finally, an enhanced chemiluminescent detection system (GE Healthcare, Piscataway, NJ, USA) was used to detect the blots. The densities of the proteins of interest were normalized to that of GAPDH.

### Immunofluorescent staining

Cytospins were thawed and fixed with 3% (v/v) paraformaldehyde in PBS for 10 min at room temperature. The slides were then washed three times for 3 min with PBS. Next, the samples were permeabilized with 1% (v/v) Triton X-100 in PBS for 10 min on ice and were washed three times. Then, they were blocked with 3% bovine serum albumin for 30 min, incubated with 0.5% phalloidin (Solarbio, Beijing, China) for 1 h and washed three times for 5 min with PBS. After that, the samples were incubated with 4′,6-diamidino-2-phenylindole (DAPI; Beyotime) in the dark for 30 min at room temperature. The slides/coverslips were washed again and mounted with Fluorescence Mounting Medium (Dako, Tokyo, Japan). The samples were stored at 4°C until imaging with a Laser Scanning Confocal Microscope (Nikon, Tokyo, Japan).

### Enzyme-linked immunosorbent assay (ELISA)

The levels of TGF-β in supernatants of primary or metastatic gastric cancer cells were detected by using ELISA kits according to the manufacturer’s instructions. TGF-β ELISA kit were purchased from MultiSciences (Lianke)Biotech Co., Ltd (Hangzhou, China).

### Statistical analysis

Each experiment was performed at least three independent times, and all data are expressed as the mean ± standard deviation. Comparisons among multiple groups were made with one-way analysis of variance followed by Tukey’s test (GraphPad Prism 7). P values <0.05 were considered statistically significant.
